# Tackling Stereochemistry in Drug Molecules with Vibrational Optical Activity

**DOI:** 10.3390/ph14090877

**Published:** 2021-08-29

**Authors:** Jonathan Bogaerts, Roy Aerts, Tom Vermeyen, Christian Johannessen, Wouter Herrebout, Joao M. Batista

**Affiliations:** 1 Department of Chemistry, University of Antwerp, 2020 Antwerp, Belgium; jonathan.bogaerts@uantwerpen.be (J.B.); roy.aerts@uantwerpen.be (R.A.); tom.vermeyen@uantwerpen.be (T.V.); christian.johannessen@uantwerpen.be (C.J.); wouter.herrebout@uantwerpen.be (W.H.); 2 Department of Chemistry, Ghent University, 9000 Ghent, Belgium; 3 Institute of Science and Technology, Federal University of Sao Paulo, Sao Jose dos Campos 12231-280, SP, Brazil

**Keywords:** vibrational optical activity (VOA), vibrational circular dichroism (VCD), raman optical activity (ROA), density functional theory (DFT), absolute configuration, pharmaceutical industry

## Abstract

Chirality plays a crucial role in drug discovery and development. As a result, a significant number of commercially available drugs are structurally dissymmetric and enantiomerically pure. The determination of the exact 3D structure of drug candidates is, consequently, of paramount importance for the pharmaceutical industry in different stages of the discovery pipeline. Traditionally the assignment of the absolute configuration of druggable molecules has been carried out by means of X-ray crystallography. Nevertheless, not all molecules are suitable for single-crystal growing. Additionally, valuable information about the conformational dynamics of drug candidates is lost in the solid state. As an alternative, vibrational optical activity (VOA) methods have emerged as powerful tools to assess the stereochemistry of drug molecules directly in solution. These methods include vibrational circular dichroism (VCD) and Raman optical activity (ROA). Despite their potential, VCD and ROA are still unheard of to many organic and medicinal chemists. Therefore, the present review aims at highlighting the recent use of VOA methods for the assignment of the absolute configuration of chiral small-molecule drugs, as well as for the structural analysis of biologics of pharmaceutical interest. A brief introduction on VCD and ROA theory and the best experimental practices for using these methods will be provided along with selected representative examples over the last five years. As VCD and ROA are commonly used in combination with quantum calculations, some guidelines will also be presented for the reliable simulation of chiroptical spectra. Special attention will be paid to the complementarity of VCD and ROA to unambiguously assess the stereochemical properties of pharmaceuticals.

## 1. Introduction

**Figure 1:**
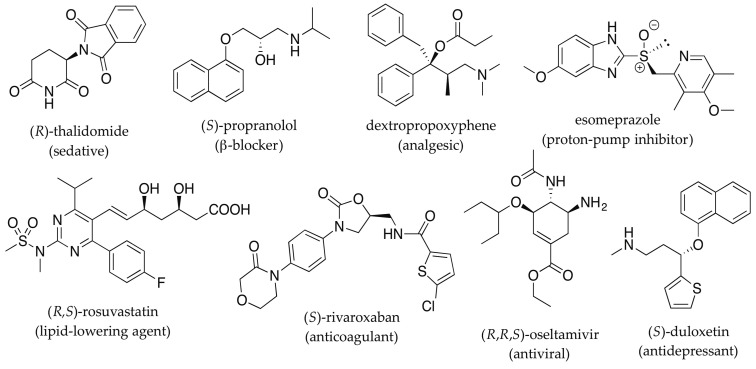
Examples of important small-molecule chiral pharmaceutical drugs.

The impact of molecular dissymmetry on the pharmacological activity of chiral drugs is a well-documented phenomenon. Seminal work by Cushny [1], Easson and Stedman [2] and Pfeiffer [3] in the first half of the 20th century has paved the ground for further developments. Chirality, however, became a more serious concern to the pharmaceutical industry following the case of thalidomide in the 1960s (Figure 1). Originally developed to treat morning sickness in pregnant women and marketed as a racemate, thalidomide (Contergan^®^; Gruenenthal, Germany) was later found to cause birth defects [4]. This iconic case also evoked the discussion on the need for enantiomerically pure drugs as opposed to racemates, since the teratogenicity was ascribed to the *S*-enantiomer (distomer) of thalidomide only [5]. Although this example has been extensively used to demonstrate the potential difference in the pharmacological and toxicological profiles of individual enantiomers (non-superimposable mirror images), problems with the animal models used and the rapid racemization observed in humans dispute the common belief that administrating the pure *R*-enantiomer (eutomer) would have prevented the occurrence of birth defects [6]. Nonetheless, this example best illustrates the importance of chirality in pharmacy and the need for reliable tools for full stereochemical elucidation and subsequent characterization of the pharmacological/toxicological profile of each stereoisomer individually before any clinical trial.

The interaction of chiral drugs with the human organism is multifactorial and marked differences between stereoisomers may be observed both in pharmacokinetics (absorption, bioavailability, distribution, metabolism, excretion) [7,8] and pharmacodynamics (receptor binding, chemical interaction, post receptor effects) parameters [4,9,10]. Apart from cases where one enantiomer is active and the other one is toxic, as in thalidomide, a number of other chiral drugs have also been demonstrated to possess distinct biological activity for its enantiomers. Examples exist where one enantiomer is more potent than the other, as in β-blocker propranolol (Inderal^®^; ICI Pharmaceuticals, London, UK), as well as where the enantiomers present different pharmacological activities, as in levo-and dextropropoxyphene (Darvon^®^, Novrad^®^, Lilly, Indianapolis, IN, USA) (Figure 1). The former is an antitussive drug while the latter is an analgesic. Chiral pharmaceuticals have also become a concern from an environmental point of view due to their improper handling in production, consumption and emission, resulting in a wide distribution of these pollutants in the environment [11]. Therefore, the stereochemical control of chiral drugs from early stages of production, through usage and disposal is pivotal in the human health care chain.

As a result, regulatory agencies around the world have stated policies regarding drug stereochemical management. The US Food and Drug Administration (FDA) published guidelines on “Development of New Stereoisomeric Drugs” in 1992 [5,12,13]. Following technological advancements in chiral separation procedures and asymmetric syntheses, the FDA put in place requirements demanding manufacturers to develop quantitative assays to evaluate the pharmacokinetics of a single enantiomer or mixture of enantiomers in in vivo samples early in drug development. The main pharmacological activities of the isomers should also be compared in in vitro systems, in animals and/or in humans. Additionally, specifications for the final product should assure identity, strength, quality and purity from a stereochemical viewpoint. As a response to stricter regulations, the pharmaceutical industry has now shifted towards developing single-enantiomer drugs as opposed to racemates [14,15]. A significant number of new single-enantiomer drugs have been developed as such (de novo), however, another common procedure includes the development of enantiomerically pure drugs from previously marketed racemates, known as chiral switches [14,16]. Enantiomerically pure drugs offer a series of advantages over racemates, such as lower doses, simplified dose-response relationships, reduced drug-drug interactions, reduced toxicity and in some cases chiral switches also allow patent protection against generic competitors [14,16].

Based on the paramount importance of the three-dimensional structures of chiral molecules for pharmacological and toxicological activities, there is an increasing demand for reliable methods to determine absolute configurations (AC) of chiral building blocks, synthetic intermediates and active pharmaceutical ingredients (API). Historically, X-ray crystallography and stereocontrolled synthesis have been the most commonly used methods for absolute stereochemistry assessment in the pharmaceutical industry [17]. Although considered the gold standard, X-ray diffraction requires quality single crystals, which may not be available at different phases of the discovery process. Additionally, the diffracting crystal selected for analysis may not reflect the stereochemistry of the bulk sample [18]. As for stereocontrolled synthesis, it depends heavily on the correct AC of the starting materials and/or catalysts as well as on the knowledge about the integrity of the chiral centers being unaltered in any subsequent chemical transformations [17]. Optical rotation (OR) that is manifested as the deviation of plane-polarized light upon interaction with a chiral non-racemic sample has also played an important role in the stereochemical characterization of drug molecules in the industry. The AC determination by means of OR, however, is possible only if the measurement has been previously calibrated using a sample of known AC. Although simple, fast and sensitive to enantiomeric excess (%*ee*) not all molecules have measurable OR. Besides, particularly when measured at a single wavelength (Sodium D line), OR may lead to highly discrepant values when changing solvent, concentration, sample purity, etc. [18]. Since OR is merely a number that carries no structural information about the sample molecules, its sole use is highly discouraged for stereochemical assignments. Despite the above-mentioned drawbacks, up until recently, OR was the only chirally sensitive technique to have a dedicated chapter in the US pharmacopeia (USP, chapter 781) [19].

As alternatives, vibrational optical activity (VOA) methods have emerged as potential complementary tools for determining the AC, conformation and enantiomeric purity of chiral pharmaceutical ingredients, directly in solution and without requiring either specific chromophores or chemical modification of the sample. VOA methods include vibrational circular dichroism (VCD) and Raman optical activity (ROA), which were first reported in the 1970s [20,21,22]. VCD is the extension of the UV-vis electronic circular dichroism (ECD) spectroscopy into the mid-infrared (IR) spectral region. VCD may be considered the chiral form of FT-IR and as such, combines the wealth of structural information inherent to vibrational spectroscopy with sensitivity to chirality. While the IR spectra of enantiomers are identical, the VCD spectra are equal in magnitude but present oppositely signed bands throughout the spectral range, with the intensities proportional to the %*ee* of the sample. ROA, on the other hand, may be considered the chiral form of Raman spectroscopy and also displays a mirror-image relationship between enantiomers over identical Raman spectra. Both VCD and ROA have undergone important technological advancements over the last 50 years that culminated in the availability of commercial instrumentation. VCD has been predominantly used for AC determinations of small organic molecules [23], while ROA has been mostly applied to the conformational analysis of water-soluble biomacromolecules [24]. As a consequence, the use of VCD is far more common in the pharmaceutical industry than ROA. ROA, however, has been reported as a sensitive diagnostic tool for the detection of higher-order structural changes of biopharmaceuticals with potential future applications in the industry [25].

VCD is currently recognized by the FDA as an acceptable method for the assignment of absolute stereochemistry [17]. Additionally, the United States Pharmacopeia (USP) contains, since 2016, two chapters dedicated to VCD (USP 39-NF34, PF 41(5), Chapters 728 and 1728), which were published following a stimuli article from 2013 [19]. In fact, VCD has been reported for AC determination of drug molecules from as early as 1992, when the (+)-enantiomer of the anesthetic agent isoflurane was assigned the (*S*)-configuration [26]. Although the vast majority of AC assignments within the pharmaceutical industry may not be made public due to proprietary issues, there are reports that virtually all major pharmaceutical companies use VCD as the preferred choice for AC assignments, either as an alternative to or complementing crystallography-based assignments [17,23,25]. Therefore, the current review article aims at covering the use of VOA methods for the assignment of AC of chiral small-molecule drugs, as well as for structural analysis of biologics with ROA. After a brief introduction of VCD and ROA theory, the best experimental practices for using these methods for stereochemical analysis will be provided along with selected examples published over the last five years. As VCD and ROA are commonly used in combination with quantum calculations, some guidelines will also be presented for the reliable simulation of chiroptical spectra. With this, we hope to complement previous literature reports [17,27] and to further demonstrate the stereochemical discriminatory power of VOA methods in order to stimulate its use by the medicinal chemistry community.

## 2. Vibrational Optical Activity

### 2.1. Theory

Similar to their non-chiral counterparts, VCD and ROA probe the vibrational energy levels of the investigated molecules. As both rely on distinct physical chemical phenomena, that is, changes in dipole moments (absorption) vs. changes in polarizability (scattering), they have proven to provide complementary information on the 3D structure of the studied molecule. In short, VCD is defined as the differential absorption of left (*A_L_*) and right (*A_R_*) circularly polarized light in the IR region of the electromagnetic spectrum. In other words, VCD is the extension of electronic CD towards vibrational spectroscopy:

Δ*A*
*= A_L_*
*− A_R_* (1)

ROA, on the other hand, is measured as the difference in Raman scattered right (*I_R_*) and left (*I_L_*) circularly polarized light by a chiral molecule:

*I_ROA_* = *I_R_* − *I_L_* (2)

Both differential quantities are directly proportional to the concentration (*C*) or the number of molecules (*N_A_*) and the enantiomeric excess (%*ee*) of the compound as shown in Equations (3) and (4). Additionally, the ROA intensities depend on the incident laser intensity (*I*_0_).

Δ*A =* Δ*ε*
*C*
*d* %*ee* (3)

*I_ROA_* = *σ_ROA_*
*I*_0_
*N_A_*
*%ee* (4)

In the equations above, Δ*ε* is the differential extinction coefficient, *d* the path length and *σ_ROA_* is the ROA cross-section. Hence, the VOA techniques provide a route to determine the %*ee* of a sample. Nonetheless, this review is focused on the application of VOA spectroscopy on the assignment of the AC of a molecule. As will be discussed in more detail later on, this requires the comparison between an experimental spectrum and quantum chemically calculated spectra. From a fundamental perspective, the VCD intensities of a vibrational transitions from the ground state (|*0>*) to an excited state (|*1>*) are determined by the electric *<0|**µ**|1>* and magnetic *<0|**m**|1>* transition dipole moments, whereas the backscattered (180°) ROA intensities are determined by the magnetic *<0|**G’**|1>* and quadrupole *<0|**A**|1>* transition polarizabilities. The VCD intensities are proportional to the rotational strengths (*R*), while the ROA intensities require the evaluation of analogue anisotropic invariants [28].

Δ*A~R = Im (<0|**µ**|1>. <1|**m**|0>)* (5)

*I_ROA_*~*12β(G’)*^2^ + *4β(A)*^2^ (6)

Thus, the fundamental complementarity of the two spectroscopic techniques lies in their dependence on different molecular properties.

### 2.2. Instrumentation

**Figure 2:**
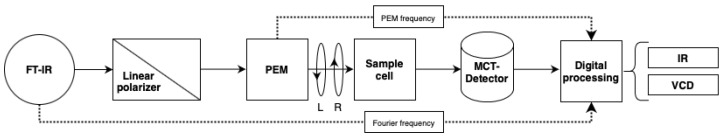
Block diagram of a FT-VCD spectrometer. See text for definitions of the used abbreviations.

**Figure 3:**
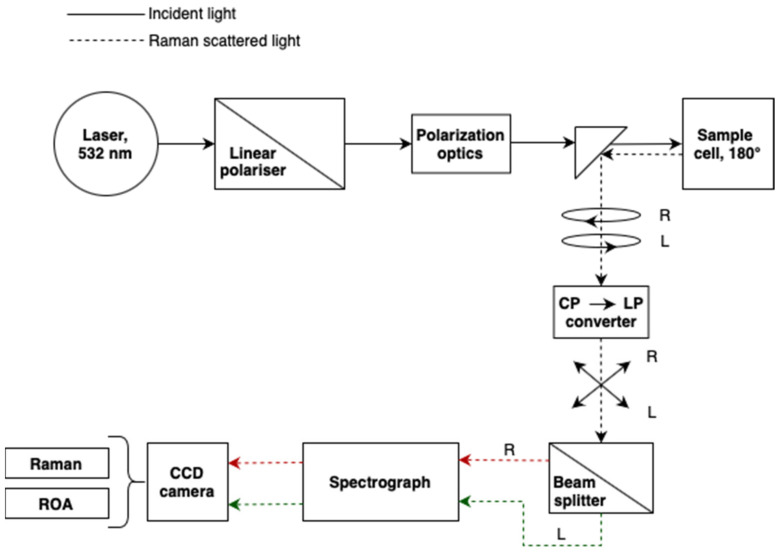
Block diagram of an SCP-ROA instrument using the backscattering (180°) strategy. See text for definitions of the used abbreviations.

VCD spectrometers became commercially available in 1997 with the introduction of the Chiral*IR*^TM^ instrument through a joint venture between Bomen and BioTools. Currently, VCD accessories and/or stand-alone instrumentation are commercialized by Bruker, Jasco and BioTools. ROA instrumentation, on the other hand, became commercially available only in 2003 when the Chiral*Raman*^TM^ spectrometer was introduced by BioTools. Today, this is still the only dedicated commercial spectrometer for the measurement of ROA. Block diagrams of the VCD and ROA instruments are depicted in Figure 2 and Figure 3, respectively. 

Modern VCD instruments make use of the Fourier-transform (FT) principle. After leaving the FT-IR spectrometer, mid-IR radiation (ca. 2000–900 cm^−1^) becomes circularly polarized by the combination of a linear polarizer with a ZnSe photoelastic modulator (PEM). The latter alternates the polarization state of the light between left and right circular polarized components at a typical frequency between 35–45 kHz. Next, the beam passes through the sample to arrive at a liquid nitrogen-cooled Mercury-Cadmium-Telluride (MCT) detector. In contrast to ordinary FT-IR spectrometers, here two interferograms arrive at the detector with different modulations, one at the Fourier frequency (ca. 1–2 kHz) and one at the PEM frequency. After processing the signal digitally (e.g., high- and low pass filtering, lock-in-amplifier) the IR and VCD spectra of the studied sample are obtained. In the Chiral*IR-2X* from BioTools, a second PEM is often placed between the sample and detector to improve the baseline quality of the experimental VCD spectrum. However, in practice, a solvent spectrum measurement under identical conditions as that of the sample is often required to be subtracted from the sample VCD spectrum to create an undistorted baseline.

The Chiral*RAMAN*^TM^ from BioTools uses the SCP-ROA setup as designed by Werner Hug [29]. Laser light with a wavelength of 532 nm first passes through a linear polarizer to remove any linear polarization impurities. Then the light passes through a train of polarization optics to remove any source that can cause polarization artifacts, either linear or circular, in the final spectrum. Then, the light is guided by prisms to the sample. Note that the instrument uses the backscattering (180°) strategy. The left and right circularly polarized light components in the scattered beam are converted into orthogonal linear polarization states (CP to LP converter). This allows the simultaneous but separate detection by using two different optical paths focused on the upper (red dotted line) and lower (green dotted line) halves of a multi-channel charged-coupled device (CCD) detector. The Raman and ROA spectra of the sample are obtained by summation and subtraction of the detected components, respectively.

### 2.3. Practical Experimental Considerations

#### 2.3.1. VCD Measurements

In order to obtain high-quality IR and VCD spectra, an appropriate selection of sample concentration, solvent and path length is required, so that the IR bands in the mid-IR region remain within 0.1–0.9 absorption units. This condition may demand the measurement of different regions of the spectrum separately in order to achieve appropriate levels of absorbance for a reliable VCD spectrum over the full mid-IR region.

As the phenomenon of CD in the infrared region is ca. 1–2 orders of magnitude smaller than that in the UV-vis [25] the measurement of a quality VCD spectrum requires larger amounts of sample (2–5 mg) and longer collection times when compared to its electronic counterpart (ECD). Typically, concentrations of 0.01–0.5 M combined with BaF_2_ sample cells having a path length ranging from 50–200 μm (for non-aqueous solutions) are most frequently used for AC determinations. Additionally, carbon tetrachloride (CCl_4_) or deuterated solvents, such as chloroform-*d*_1_ (CDCl_3_), acetonitrile-*d*_3_ (ACN-*d*_3_), methanol-*d*_4_ (CD_3_OD) and dimethylsulfoxide-*d*_6_ (DMSO-*d*_6_) are used to maximize the spectral window without solvent interference. If there is a need for the measurement of aqueous solutions, sample cells with CaF_2_ windows and path lengths of 5–15 μm are required. Also in this case the use of the deuterated solvent (D_2_O) is preferred. IR and VCD spectra are collected simultaneously with a spectral resolution usually in the range of 4–8 cm^−1^. Data are collected using blocks of variable time lengths (or a variable number of scans), which are then averaged to yield the final spectrum. The total collection time of a VCD spectrum varies roughly from 1 to 12 h to achieve a desirable signal-to-noise (S/N) ratio. Finally, the VCD spectrum baseline is corrected either by taking the half difference of the VCD spectra of both enantiomers (recommended option) or by subtraction with the VCD spectrum of the corresponding racemate or solvent measured at similar conditions.

#### 2.3.2. ROA Measurements

For recording high-quality ROA spectra, highly concentrated samples (0.1–1 M), are necessary, as the ROA signal is intrinsically very weak (~10^−3^ with respect to the parent Raman signal) [20,23]. Very often concentrations around 30–50 mg/mL are also found in the literature. Due to the smaller volumes needed in ROA cells (40–50 μL), the amount of compound required is similar to that of VCD measurements (i.e., 2–5 mg). To record ROA signals and improve the S/N ratio, relatively long measurement times are needed, ranging between 12 h and a couple of days. Depending on the sample composition and concentration, the laser power is typically in the order of 250–800 mW. This should be set to prevent the saturation of the CCD detector. For samples with purity below 98% (as determined by HPLC), background fluorescence may become an issue. In order to reduce fluorescence levels, photobleaching may be required, that is, irreversible conversion of impurities to non-fluorescent forms by placing the sample in the laser beam for a couple of hours prior to the actual measurement. Protocols involving kinetic quenchers also exist to reduce strong fluorescent backgrounds [30]. The same types of solvents used for VCD can be employed during ROA measurements. Additionally, aqueous solutions (e.g., buffer systems) are highly compatible with ROA, which might be necessary for solvating certain compounds (proteins, carbohydrates, etc.) in a sufficiently high concentration. Measurements are carried out in individual Raman and ROA scans which are summed not averaged. Optionally, the resulting ROA spectrum can be smoothed by applying a Savitsky-Golay filter (typically third order, frame length 9). In contrast with VCD where the enantiomer and/or racemate is measured for baseline corrections, the commercial ROA instrument is built to automatically measure the optically created virtual enantiomer to correct the spectral baseline [31]. However, baseline offsets sometimes still occur and can be corrected using a Savitsky–Golay filter (3–4th order, frame length 201–401) or a Fourier-transform filter during the post-processing step. For the Raman spectrum, a solvent measurement should be recorded and subtracted from the sample spectrum. It also allows the identification of the spectral range that is not obscured by the solvent. A subsequent Eilers–Boelens baseline correction is advised to remove fluorescent backgrounds from the final Raman spectrum [32].

### 2.4. Computational Workflow for AC Determination

Once experimental VOA spectra have been recorded, the extraction of the absolute configuration or the conformational population of a given molecule directly from observed data is not possible most of the time, with some notable exceptions (see subsequent sections). For this reason, quantum chemical spectral predictions are essential for AC determination and conformational analysis. Due to the fundamental similarity between the two methods, the computational workflow for both VCD and ROA applications is essentially identical and thus mostly based on the exact application instead of the spectroscopic method itself. However, it is important to stress that quantum chemical predictions of VCD spectra remain easier than that for ROA, which requires longer computation times and higher quality basis sets.

**Figure 4:**
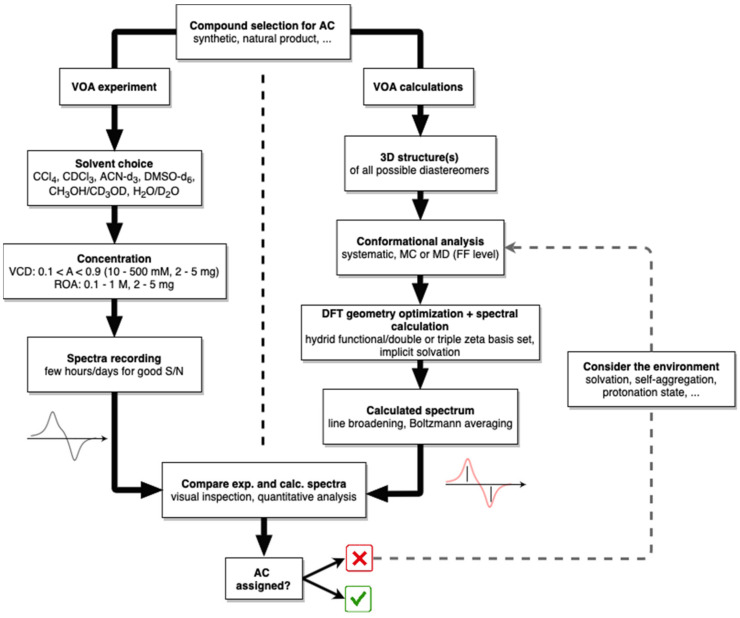
Typical workflow for AC determinations using VOA techniques. See text for more details.

A typical computational workflow for AC determination with VOA methods is presented in Figure 4. At this point, for the full structural characterization of a compound, it is assumed that the 2D-molecular structure has been secured through mass spectrometry (MS) and nuclear magnetic resonance (NMR) analysis. Furthermore, when the compound has multiple chiral centers, it can be advantageous to use NMR spectroscopy and/or X-ray crystallography to elucidate the relative configuration, limiting the number of possible diastereoisomers to be considered in the VOA analysis. Firstly, a 3D structure for one enantiomer of each possible diastereoisomer is extracted from a database or generated using one of many computational chemistry libraries or programs. The initial choice of which enantiomer will be considered is not relevant, since, at the end of the workflow, inversion of the signs of the intensities will yield the spectral data for the opposite enantiomer.

A conformational search is then initiated using the 3D structures. Typically, this is performed on a force field (FF) level using a systematic search, Monte Carlo (MC) or molecular dynamics (MD) based approach. This step is non-trivial when tackling larger and/or more complex systems, where exclusion of a single or a group of conformers can prove detrimental to the quality of the predicted spectrum. The vast number of different conformational analysis programs available, both commercially and open source, indicates that there is no single perfect method for the system under investigation. Considering the low computational cost associated with force fields, one can instead collect the conformations generated by multiple programs/algorithms to cover the shortcomings of a specific method. The geometries of the different conformers are subsequently optimized using density functional theory (DFT) followed by frequency calculations. The latter will tell whether the different conformers are optimized to their local minimum by the absence of imaginary frequencies. The VCD and/or ROA intensities for the vibrations of the resulting conformers are then calculated. VCD must be calculated using the same level of theory employed for geometry optimization, while for ROA either the same of different levels can be used.

DFT calculations are performed by combining a functional with a basis set. The standard functionals for spectral predictions are B3LYP or B3PW91. The solvent is often considered implicitly by using the polarizable continuum model (PCM). For VCD, 6-31G(d) is generally recommended by the community as the minimal basis set, while larger basis sets with diffuse functions (e.g., 6-31++G(d,p)) are required to obtain calculated ROA spectra of sufficient quality. For ROA calculations the so-called rDPS basis set has been designed and is often used as a low-cost alternative [33]. The exact choice of basis set should be based on the available computational resources along with the size of the system, as the computational time increases with the size of the basis set and the number of electrons/atoms. The cost associated with the prediction of the Boltzmann spectrum can be lowered when necessary by only considering the conformers obtained from the conformational analysis within a low-energy window of, for example, 5–15 kcal/mol, with the energy determined on a force field, or <3 kcal/mol for DFT-optimized structures at a small basis set level. For systems of large size, fragmentation-based approaches such as Cartesian tensor transfer (CTT) [34] or molecule-in-molecules can be applied [35].

Finally, the predicted line spectra are broadened using a Lorentzian band shape with a full-width at half-maximum of 10–15 cm^−1^ or 15–20 cm^−1^ for VCD and ROA, respectively. The broadened spectra of individual conformers are then weighted according to their relative population determined by the Boltzmann distribution and linearly combined to yield the final calculated spectra for each pair of enantiomers. The Boltzmann weights are calculated using relative zero-point corrected energies, relative enthalpies or relative Gibbs energy, according to which fits best with the experiment. However, for a robust AC assignment, this choice may not have an impact on the assignment.

The AC of the compound is established by comparison of the experimental spectrum and the calculated spectra of all possible ACs. A scaling factor (0.95–1.05) on the calculated frequencies will often be applied to account for systematic errors in the vibrational frequencies caused by the harmonic approximation and the use of a limited basis set. The similarity between experimental and calculated spectra can be determined by either visual inspection or various quantitative methods that make use of similarity measures [36,37,38,39]. When none of the predicted spectra match the experimental spectrum to a satisfying degree, after ruling out errors in the structure elucidation one should either increase the basis set size (if of limited size) or consider the environment of the molecule. The latter can be in the form of different protonation states, explicit consideration of the solvent or molecular aggregates. The inclusion of dimers or explicit solvation can be challenging for non-experts. Recent work by the Merten group, however, has shown that for specific functional groups systematic approaches can be used in a straightforward manner to account for this [40,41,42,43].

As demonstrated above, the workflow for AC determination with VOA also allows the assessment of the predominant conformations in solution. Additionally, due to the exceptionally high sensitivity of VOA spectra to the conformational population of the compound in question, a high similarity between experimental and computational spectra can only be obtained if all the important conformations are considered. When the AC of a compound is already known, its dominant conformers in solution can be established by comparing the broadened conformational spectra directly with the experimental spectrum. The full conformational population can also be established by fitting the weights for the linear combination of the conformational spectra, such that the resulting averaged spectrum generates the highest similarity with the experimental spectrum. Precautions should be taken, however, as one could easily fit these weights to any random spectrum if the number of conformer spectra is very large. When the number of conformers is too extensive, clustering the conformers on their geometrical features and extracting representative structures from these clusters can be considered for spectral predictions [44].

## 3. Application Examples

In this section, selected examples of the application of VOA techniques for the stereochemical investigation of molecules of pharmaceutical interest will be presented. The first two examples will demonstrate the power of either VCD or ROA to assess the stereochemistry of the molecules, and then the advantages of combining both techniques will be presented. Finally, the use of ROA to determine conformational changes of biologics will be discussed.

### 3.1. VCD Example

**Figure 5:**
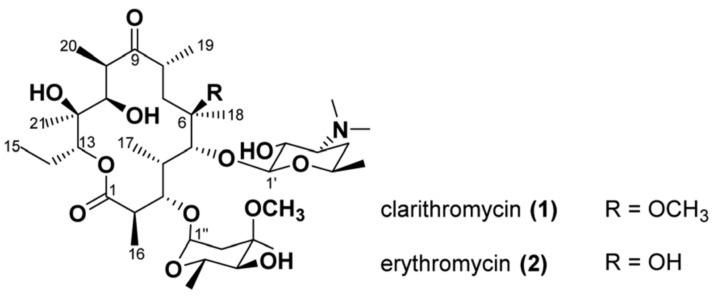
Chemical structures of clarithromycin and erythromycin. Reprinted with permission from ref. [45]. Copyright 2020 Royal Society of Chemistry.

The first example by Demarque and co-workers [45] involves the use of IR and VCD for the stereochemical characterization of two macrolide antibiotics, clarithromycin and erythromycin, directly in solution-state. These compounds present complex and flexible structures with multiple chiral centers that impose challenges for the use of vibrational chiroptical spectroscopy. Additionally, the sole structural difference is the presence of a methoxy group at C-6 in clarithromycin and a hydroxyl group at the same position in erythromycin (Figure 5). Regarding stereochemistry, the relative configurations of the stereogenic centers C-2 to C-5, C-10 and C-11 of the macrolides can be obtained from NMR spectra by analysis of the *J*-coupling constants. However, for C-6, C-8, C-12 and C-13 no such coupling constants are available. Therefore, this example demonstrates the structural and stereochemical discriminatory power of VCD even in suboptimal conditions. By comparing experimental and simulated IR/VCD data not only was the assignment of the absolute configuration of these macrolides possible, but also the identification of their predominant solution conformations. 

**Figure 6:**
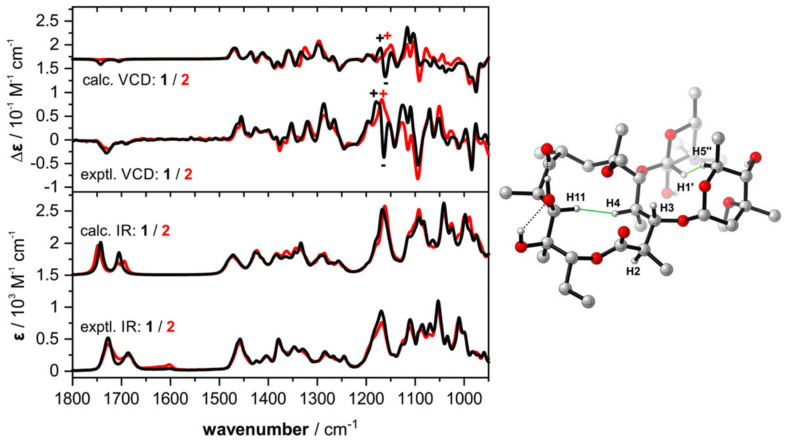
((**Left**) Comparison of experimental and calculated IR and VCD spectra of clarithromycin (1) and erythromycin (2). (**Right**) Lowest-energy conformer of clarithromycin adopting a “folded-out” conformation. Reprinted with permission from ref. [45]. Copyright 2020 Royal Society of Chemistry.

**Figure 7:**
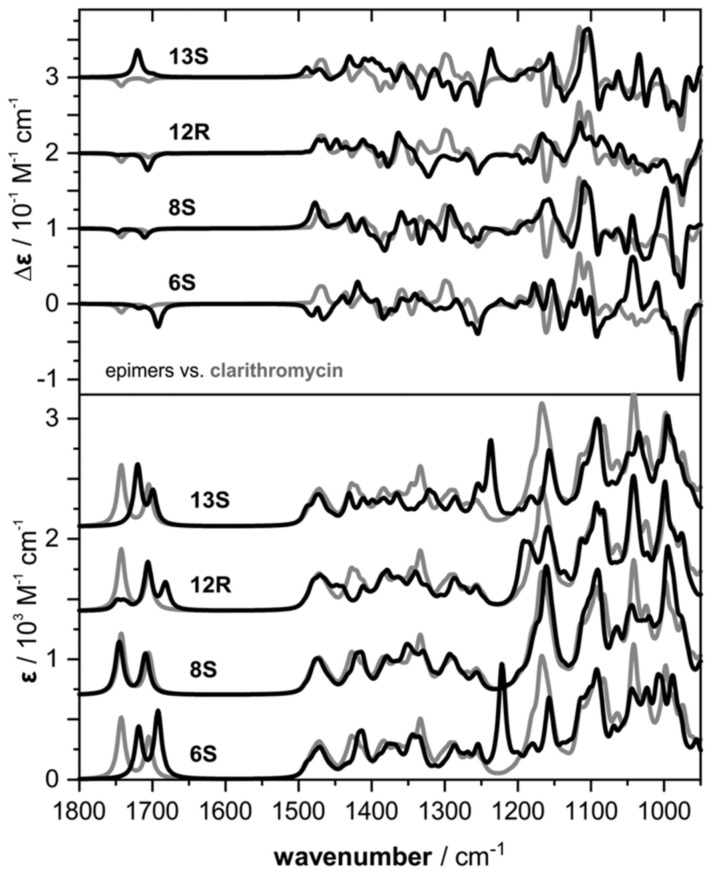
Comparison of the calculated IR and VCD spectra of the epimers 6*S*-, 8*S*-, 12*R*- and 13*S*-clarithromycin with the spectra computed for the actual configuration of clarithromycin (grey line), as depicted in Figure 5. Reprinted with permission from ref. [45]. Copyright 2020 Royal Society of Chemistry.

Initially, as expected, the comparison of the experimental IR spectra obtained in CDCl_3_ for clarithromycin and erythromycin indicated higher similarity rendering them almost indistinguishable. Their VCD spectra, however, despite an overall similarity, presented distinct spectral features at around 1175 cm^−1^ that can be used to tell them apart (+,− pattern for clarithromycin and + pattern for erythromycin) (Figure 6). In order to interpret the experimental data, DFT spectral simulations were performed for both structures. The computational approach included a multi-step comprehensive Monte Carlo conformational search on force-field level (MMFF) followed by geometry optimization steps and frequency calculations at the B3LYP/6-31G(2d,p)/IEFPCM(CHCl_3_) level of theory. Interestingly, in spite of the structural intricacy of both compounds, only a few conformers were found to significantly populate the samples (one for clarithromycin; and four for erythromycin), with a predominance of the so-called folded-out conformation. As for the sugar molecules attached to C-3 and C-5, stable chair conformations were proposed based on observed NOE contacts. The DFT-simulated spectra correctly reproduced the experimental data and allowed the identification of the vibrational modes responsible for the visual differences in the VCD spectra of clarithromycin and erythromycin around 1150–1200 cm^−1^. These modes included the C-1–O stretching motion coupled to C–H bending motions at C-2 and C-13 that were common to both macrolides (positive VCD bands at 1178 and 1168 cm^−1^), as well as strong contributions of the C–O–C stretching vibration of the methoxy group (negative VCD band at 1164 cm^−1^), that is characteristic for the spectral signature of clarithromycin. Further IR/VCD studies on derivatives of clarithromycin with the sugar at C-3 replaced with either OH or a 2-phenylacetic acid ester resulted in little spectral differences. These results indicated only small contributions of the sugar moieties to the overall spectral pattern with the main characteristic spectral signatures localized on the macrocycle. Finally, comparisons of calculated IR/VCD spectra for C-6, C-8, C-12 and C-13 epimers of clarithromycin demonstrated significant spectral differences (Figure 7) that showcase the enhanced sensitivity of VCD to the stereochemistry of individual chiral centers, including challenging non-hydrogenated stereocenters. These results also suggest that an experimental differentiation between these epimers should be possible from a single IR/VCD measurement. 

### 3.2. ROA Example

Whereas VCD has been offering a complementary route to structure elucidation and absolute configuration analysis, especially when classical methods (X-ray and NMR) cannot be applied, ROA has for historical reasons been mainly applied to structural studies of biomacromolecules. Although the added value of ROA has now been clearly proven, applications of ROA for absolute configuration determination still remain scarce. Below follows a successful example of the use ROA for small-molecules absolute stereochemistry investigation.

An interesting ROA study involves the discrimination of the four possible stereoisomers of the synthetic precursor 1-*t*-butyloxycarbonyl-3-triethylsilyloxy-4-phenyl-2-azetidinone (1-BOC-3-TES-4-Ph-azetidin-2-one), used for the production of taxol, from baccatin III [46].

**Figure 8:**
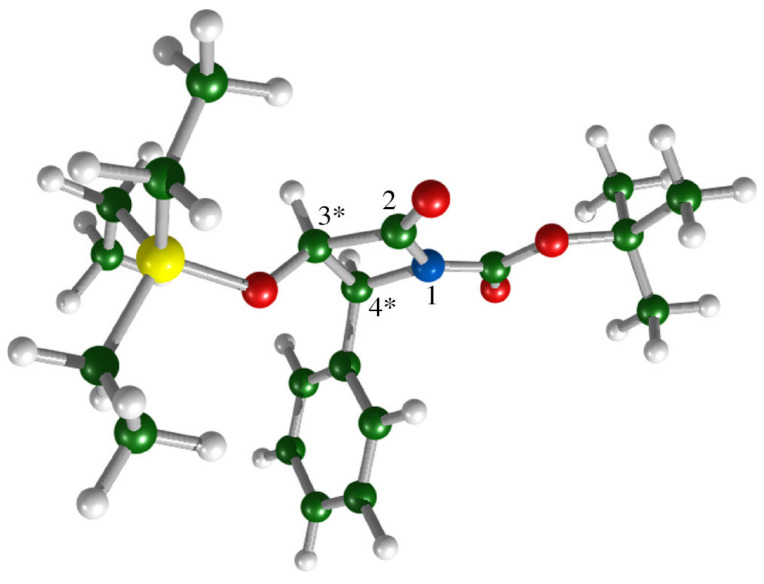
Structure of the (3*R*,4*S*)-diastereoisomer of the 1-BOC-3-TES-4-Ph-azetidin-2-one precursor, with labeled numbering of the azetidine ring. Chiral carbon atoms are marked by asterisks. Reprinted with permission from ref. [46].Copyright 2017 American Chemical Society.

Taxol is one of the most powerful natural anticancer agents and one of the top-selling drugs used in the treatment of breast, lung and ovarian cancers [47]. Taxol can be isolated from the bark of the Pacific yew tree (*Taxus brevifolia*), but the tree is rather rare and the yield of the compound is extremely low. This, together with the high demand for taxol, resulted in a tremendous effort to obtain this molecule synthetically, with a total synthesis of the product first reported in 1994 [48,49]. However, the synthetic route proposed is not economical and is now replaced by semi-synthetic approaches based on chemical modifications of natural taxoids such as baccatin III with 1-BOC-3-TES-4-Ph-azetidin-2-one [50]. As the azetidine ring bears two chiral centers, four (2^2^) possible stereoisomers can exist, but only the (3*R*,4*S*)-diastereoisomer shown in Figure 8 can be used for functionalization.

In their study, Profant and co-workers [46] investigated the performance of ROA spectroscopy in distinguishing the four possible stereoisomers of the synthetic precursor. To this end, the experimental ROA spectra for two samples marked as A and B with different, but unknown, absolute configurations dissolved in MeOH to a concentration of 50 mg/mL, were recorded and compared with calculated data for the (3*S*,4*S*)- and (3*R*,4*S*)-diastereoisomers. The spectra of their enantiomers display mirror-imaged ROA properties and can be obtained by multiplying the spectral intensities of each diastereoisomer by (−1). In this case, ROA was chosen to avoid limitations due to the low solubility of the compound in the traditionally used solvents for AC determination and to increase the spectral window from 950 cm^−1^, the common lower limit of the VCD MCT detector for CDCl_3_ measurements, down to 200 cm^−1^.

**Figure 9:**
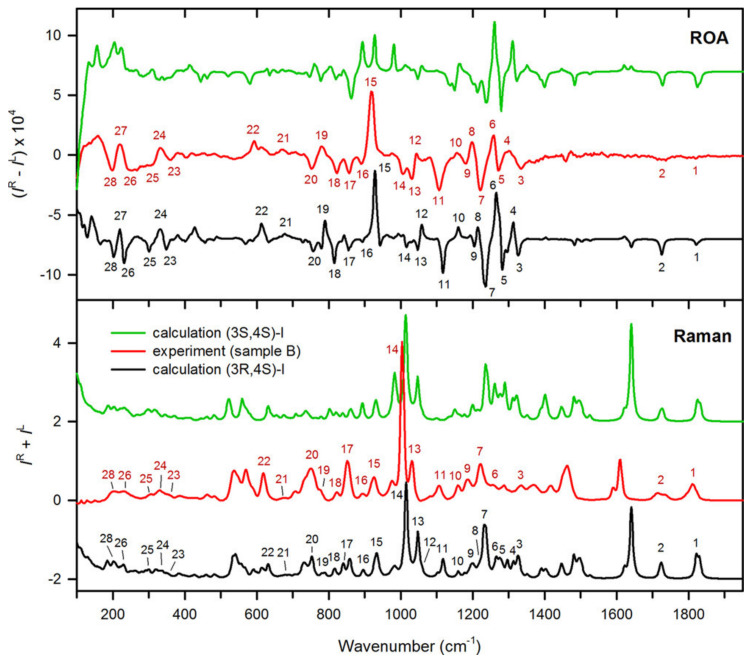
Comparison of the experimental (sample B) and simulated Raman and ROA spectra of the (3*R*,4*S*)- and (3*S*,4*S*)-diastereoisomer of the 1-BOC-3-TES-4-Ph-azetidin-2-one precursor studied. Reprinted with permission from ref. [46]. Copyright 2017 American Chemical Society.

The experimental spectra for sample B and the calculated data for the diastereoisomers are compared in Figure 9. By combining the ROA experiment with theoretical simulations of the spectra, the authors were able to assign the absolute configurations of the test samples A and B as (3*S*,4*S*) and (3*R*,4*S*), respectively, with a high degree of confidence. It was explicitly noted that whereas the chirality at C-4 could be determined relatively reliably, the assignment of the absolute configuration at C-3 was more challenging, as there were only a limited number of weak ROA bands connected to it. Nevertheless, the correspondence between the experimental and calculated spectra was sufficient to secure the complete AC assignment.

### 3.3. VCD + ROA Examples

#### 3.3.1. Galantamine

In order to further demonstrate the versatility of the VOA techniques and their complementarity for AC determinations, De Gussem and co-workers [51] recently reported a combined experimental and theoretical study in which three solution-based methods, NMR, IR/VCD and Raman/ROA were applied to the same sample. As the reference system, the authors chose the chiral alkaloid galantamine. This drug is approved by the EMA (European Medicine Agency) and the US FDA as a competitive inhibitor of acetylcholinesterase and as an allosteric modulator of the nicotinic acetylcholine receptor that has proven effective in the treatment of mild-to-moderate Alzheimer’s disease [52,53,54,55,56].

**Figure 10:**
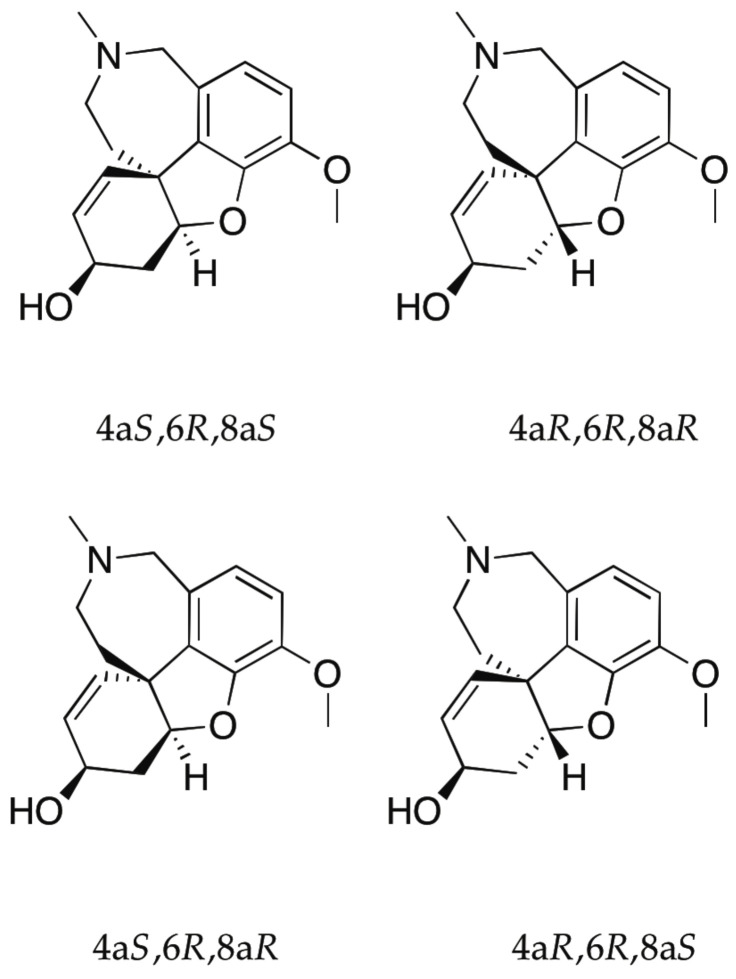
Chemical structures of the four diastereoisomers possible for galantamine.

Galantamine contains three chiral centers, thereby increasing the number of stereoisomers possible (Figure 10) and consequently the level of complexity compared to that of the taxol precursor discussed above. Based on the possibility of significantly different biological activities for the stereoisomers of medicinally active molecules, knowledge of the relative and absolute configuration of compounds such as galantamine is of utmost importance.

**Figure 11:**
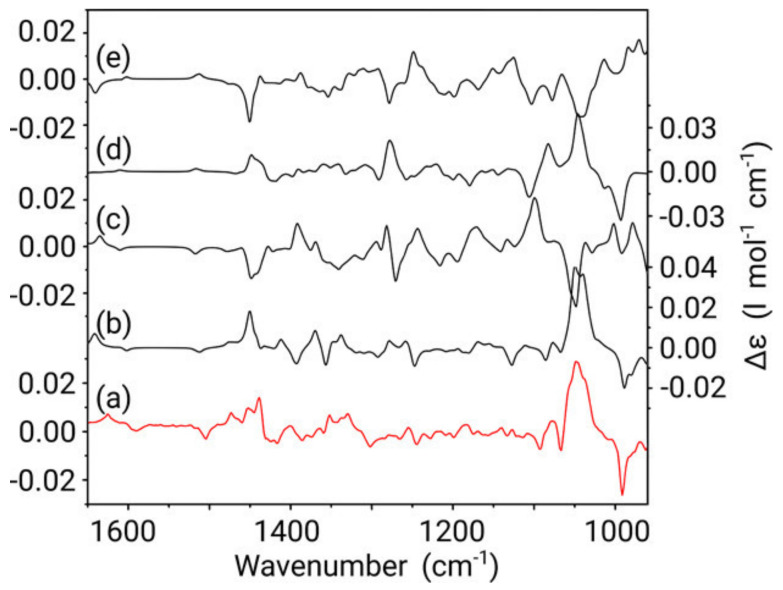
Comparison between the experimental VCD spectrum (measured in CDCl_3_) of galantamine (**a**) and Boltzmann weighted calculated VCD spectra of the 4a*S*,6*R*,8a*S* (**b**), 4a*S*,6*R*,8a*R* (**c**), 4a*R*,6*R*,8a*S* (**d**) and 4a*R*,6*R*,8a*R* (**e**) configurations of the molecule. *Y*-axis labels are placed alternating left/right to avoid congestion. Reprinted with permission from ref. [51]. Copyright 2019 American Chemical Society.

The experimental and calculated VCD spectra for the different diastereoisomers of galantamine are depicted in Figure 11. Based on the similarities of the calculated VCD spectra for the diastereoisomers, the authors concluded that VCD could be used to assign the correct enantiomer of galantamine only with prior knowledge on the relative configuration obtained from NMR spectroscopy.

**Figure 12:**
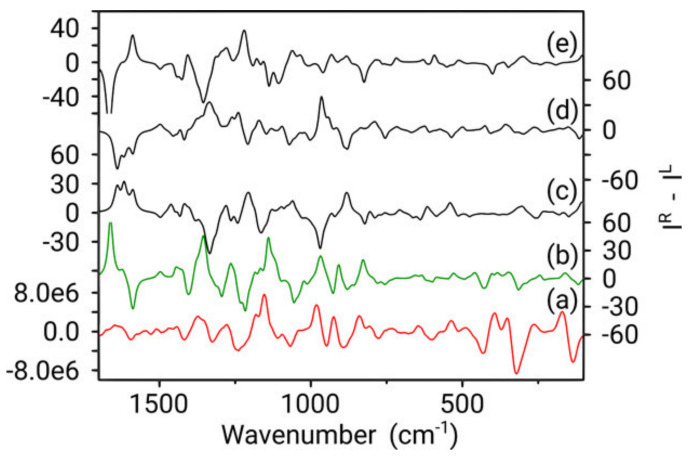
Boltzmann weighted calculated ROA spectra for 4a*S*,6*R*,8a*S* (**b**), 4a*S*,6*R*,8a*R* (**c**), 4a*R*,6*R*,8a*S* (**d**) and 4a*R*,6*R*,8a*R* (**e**), compared with the experimental ROA spectrum (measured in CHCl_3_) (**a**). *Y*-axis labels are placed alternating left/right to avoid congestion. Reprinted with permission from ref. [51]. Copyright 2019 American Chemical Society.

The ROA spectra in Figure 12 reveal a different situation. It becomes immediately apparent that the predicted spectrum for the 4a*S*,6*R*,8a*S* configuration (green spectrum) of galantamine represents the best comparison to the experiment. Except for an intensity overestimation of the couplet between 1660 and 1700 cm^−1^ and the positive band at 1350 cm^−1^, most bands from this configuration can be readily assigned in terms of shape, position and intensity to the experimental spectrum. This is not the case for any of the other three possible configurations and neither does an assignment of the opposite enantiomer seem appropriate. Therefore, from a fast qualitative analysis, the AC of galantamine could be assigned solely with ROA.

#### 3.3.2. Artemisinin and Derivatives

Recently, studies on the artemisinin scaffold were initiated to shed more light on the direct applicability of VOA in structure elucidation of chiral compounds containing a large number of chiral centers and thus an increased amount of possible diastereoisomers (2^x^ with x = number chiral centers) that needs to be distinguished [57,58].

**Figure 13:**
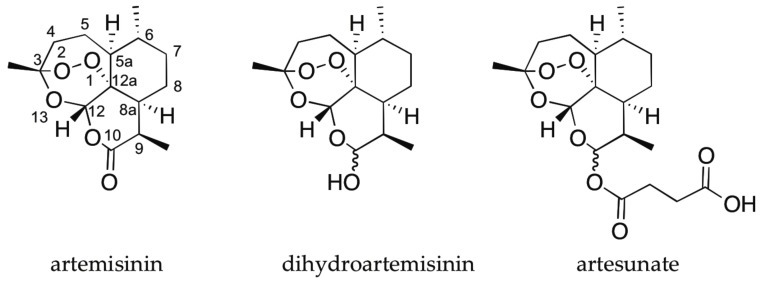
Chemical structures of artemisinin, dihydroartemisinin and artesunate. Reprinted with permission from ref. [58]. Copyright 2020 Royal Society of Chemistry.

Artesunate, an artemisinin derivative, is recommended by the world health organization (WHO) as the first choice in the treatment of severe malaria tropica (caused by the *Plasmodium falciparum* parasite) for adults, children and pregnant women as it provides a rapid clinical effect in patients [59]. The importance of the compound is highlighted by the Nobel prize awarded to Tu Youyou in 2015 for the elucidation of artemisinin and efforts to achieve effective scalable synthetic routes for the synthesis of the compound [60,61,62]. Chemically, artemisinin is a sesquiterpene lactone that has seven chiral centers of which two are locked together in an endoperoxide bond. The latter is responsible for the activity of the compound as it creates free radicals that inhibit the function of the *Plasmodium* parasite. With the need for a more hydrophilic derivative, artemisinin was converted into artesunate, by first reducing it to dihydroartemisinin with subsequent reaction with succinic anhydride (Figure 13).

**Figure 14:**
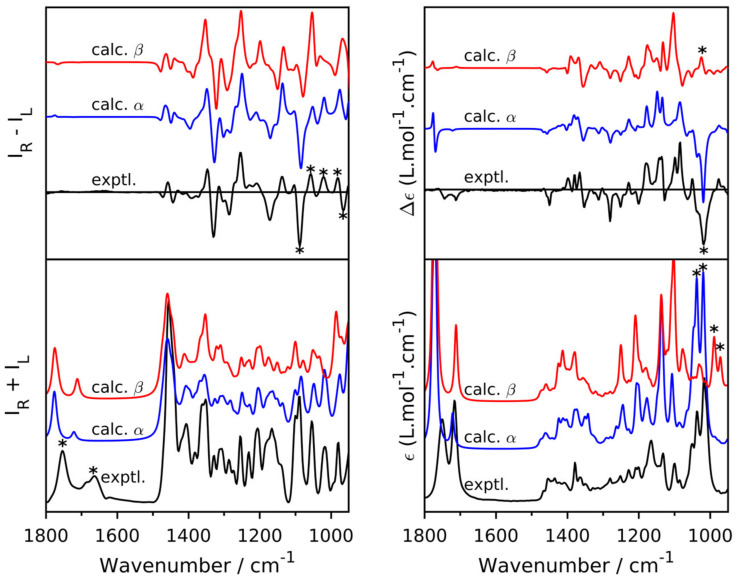
Comparison of experimental and calculated Raman/ROA (**Left**) and IR/VCD spectra (**right**) of artesunate. The asterisks (*) in the ROA, VCD and IR spectra indicate visual assignment to the α form. The (*) in the Raman spectrum (bottom left) indicate the typical overestimation of carbonyl stretch vibration in QM calculations. Reprinted with permission from ref. [58]. Copyright 2020 Royal Society of Chemistry.

Upon the conversion of artemisinin to artesunate, an additional chiral center is created at C-10 and therefore, artesunate can exist in two possible epimers, referred to as α and β depending on whether the succinic anhydride tail has, respectively, an equatorial or axial orientation. As mentioned above, full stereochemical characterization is of utmost importance in pharmacy and hence elucidation of the chiral center at C-10 is needed when all other chiral centers are already assigned. In order to determine the chiral center at the C-10 position, Bogaerts and co-workers [58] applied VOA spectroscopy (100 mg/mL sample in CDCl_3_ for ROA, 40 mg/mL for VCD) combined with DFT calculations (B3PW91/6-31++G(d, p)) on both possible C-10 epimers. The experimental and calculated VOA spectra are shown in Figure 14. Based on a visual agreement, the experimental VCD and ROA spectra could be assigned to the α-form (highlighted with *). This assignment was strengthened by a quantitative analysis using a regular overlap integral, displaying a significantly larger value for the correct epimer (overlap of 84.0% vs. 56.5% for ROA and 87.8% vs. 35.6% for VCD). Although in theory, conventional vibrational spectroscopy should be able to distinguish between diastereoisomers, in practice, this is sometimes difficult. The calculated Raman spectra of the two epimers show a highly similar pattern making it impossible to assign one of them unambiguously to the experiment (overlap of 91.5% vs. 89.7%), while IR presents a better discriminatory power (overlap of 90.6% vs. 61.2%). The fact that the VOA spectroscopies show distinct spectral patterns for the two epimers studied highlights the added value of using vibrational chiroptical spectroscopy for this stereochemical challenge. The authors also demonstrated the synergism between the two VOA techniques for the AC determination. When VCD and ROA were combined, the AC assignment of artesunate starting from all the possible 64 diastereoisomers was achieved. Even though both ROA and VCD individually showed the highest agreement for the correct diastereoisomer, when combined, the two spectroscopic techniques led to an even more reliable assignment.

### 3.4. ROA Examples on Biologics

While VCD has established itself firmly in the field of AC determination, historically, research in ROA has focused on studying bio(macro)molecules. Following a phase concerned with fundamental studies and instrumental advancements [63,64,65], the ROA field took a turn around 1990 towards the structural characterization of biologically relevant systems after several promising measurements of proteins in aqueous solution [66,67]. The interest in using Raman-based spectroscopies for studying biochemicals lies in the Raman transparency of aqueous solution samples, which is not the case for IR-based spectroscopies. Hence, numerous empirical structural studies on peptides and (glyco)proteins, carbohydrates, nucleic acids and intact viruses have been conducted during the 1990s [66,68,69,70,71,72,73,74,75,76,77,78,79,80,81,82,83,84,85,86,87,88,89]. It has been demonstrated that ROA is highly sensitive towards the protein secondary structure, each structural type displaying distinct spectral features [75,76,90,91].

From the establishment of the first empirical structure-spectrum relationships of peptides and proteins, the ROA technique has been positioning itself in a complementary manner among the existing techniques: high-resolution X-ray diffraction and multi-dimensional solution NMR. For instance, growing crystals or extracting NMR conformational ensembles for proteins that fall under the intrinsically disordered proteins (IDPs), a general descriptor for systems that have been referred to as flexible, mobile, partially folded, natively denatured, natively unfolded, chameleon, disordered, etc. Ref. [92] is very difficult to impossible [93,94]. These types of proteins have higher structural flexibility, rendering them very dynamic, hampering crystal growth and/or straightforward NMR analyses. Recording the ROA spectra of these types of proteins, on the other hand, is routine and their structure-spectrum relationship has been extensively investigated [91,93,94,95,96,97,98,99]. As such, ROA research has demonstrated the importance of the residual presence of left-handed poly-L-proline type II (PPII) helix in IDPs [94,96,100,101]. As these types of proteins can play an important role in (neurodegenerative) diseases, ROA can help with solving the structure-function puzzle, eventually elucidating mechanisms of actions for drug development, treatments and disease prevention. In this section, we showcase two recent studies where ROA assists in tackling pharmacological issues, namely, the structural behavior of α-synuclein in different environments and the in vivo detection of Alzheimer’s disease by means of ROA.

#### 3.4.1. α-Synuclein in Different Environments

**Figure 15:**
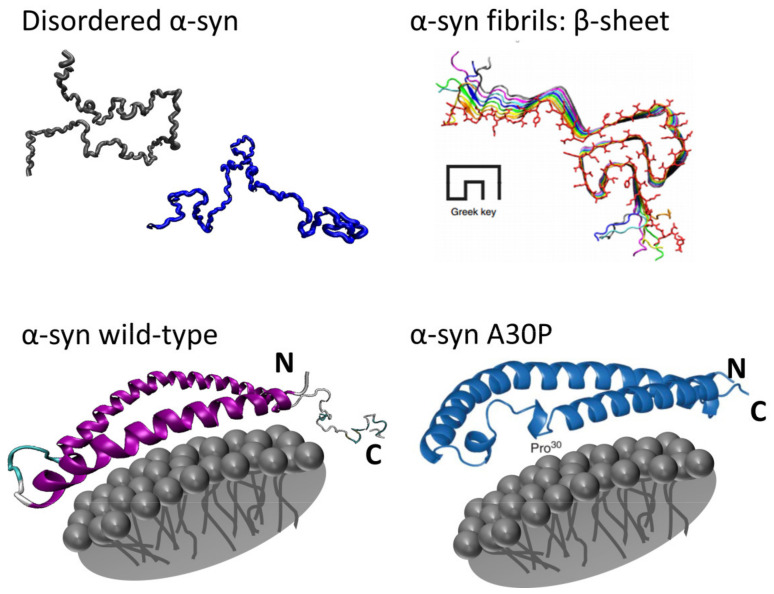
The different structures α-synuclein (α-syn) can take. Reprinted with permission from ref. [104]. Copyright 2017 John Wiley and Sons.

α-synuclein is a protein consisting of 140 amino acids and is mainly expressed in presynaptic nerve terminals [102]. This protein has a structurally chameleonic nature: it behaves as an IDP when in monomeric form, adopts an α-helical structure when in contact with membranes and has the ability to aggregate to form fibrils (Figure 15). In its monomeric form, at times by interacting with cellular membranes, α-synuclein can play a large variety of roles in the cellular organelles of the neurons [103]. Unfortunately, its ability to form fibrils has been found to be associated with the pathogenesis of neurodegenerative diseases such as Parkinson’s disease (PD) and dementia with Lewy bodies (DLB). Within the brain of patients with PD and DLB, the presence of fibrillar aggregates that (partially) exist out of insoluble α-synuclein has been found (Lewy bodies). 

**Figure 16:**
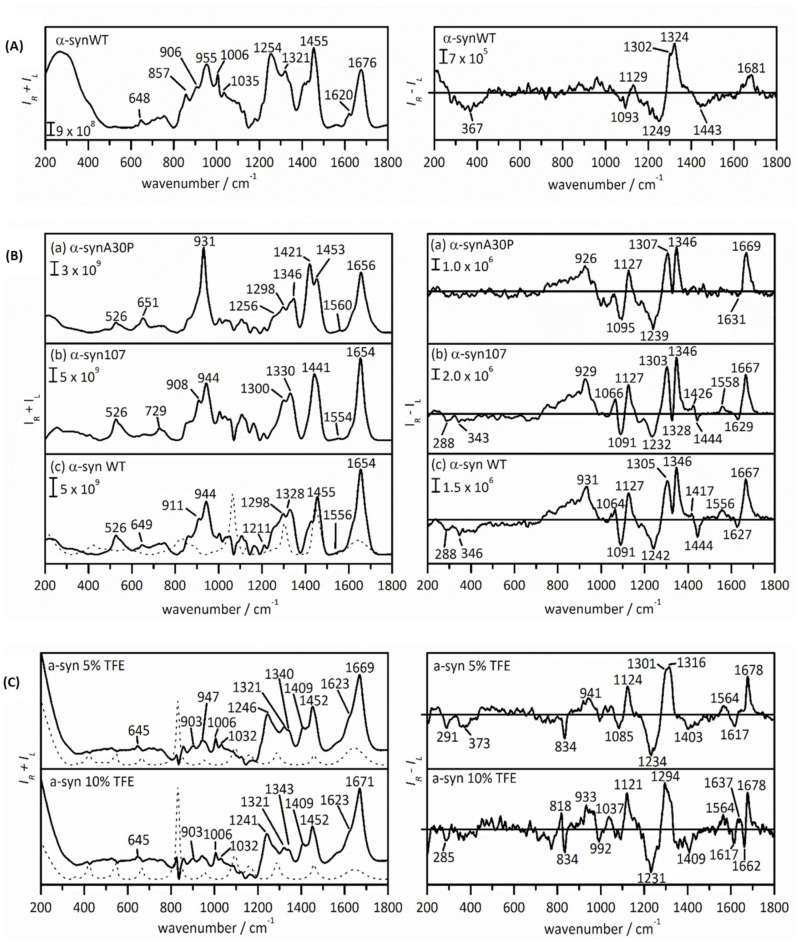
The Raman (left) and ROA (right) spectra of human wild-type α-synuclein in aqueous solution (**A**); the α-synuclein A30P variant (**a**), the α-synuclein 107 variant (**b**) and human wild-type α-synuclein (**c**), all in a high concentration of sodium dodecyl sulphate solution (**B**); wild-type α-synuclein in 5% (top) and 10% *v*/*v* (bottom) 2,2,2-trifluoroethanol (TFE) in demineralised water (**C**). The dashed lines in the Raman spectra are the corresponding solvent background spectrum. Reprinted with permission from ref. [104]. Copyright 2017 John Wiley and Sons.

To deepen the general understanding of the structural behavior and dynamics of α-synuclein and their implications in its biological effects, Mensch and co-workers [104] employed ROA to investigate the protein under several conditions. In aqueous solution the protein returns ROA (and Raman) spectral profiles highly indicative of an IDP “conformation”: a broad ROA amide I band with a maximum at 1680 cm^−1^, a broad maximum at 1324 cm^−1^ and a −/+ couplet at 1093/1129 cm^−1^ (Figure 16). These characteristic bands are, besides a disordered structure, indicative of PPII helical structure, as mentioned before. This result lies in line with NMR studies where in vitro α-synuclein adopts a disordered structure with tendencies to adopt PPII structure [105,106].

Upon mixing of α-synuclein with membrane mimicking sodium dodecyl sulfate micelles, the resulting ROA (and Raman) spectra changed drastically, displaying spectral profiles of protein dominated by the α-helical conformation (Figure 16): a −/+ couplet at 1627/1667 cm^−1^, a −/+/+ pattern at 1242/1305/1346 cm^−1^ and a −/+ couplet at 1091/1127 cm^−1^. The positively charged N-terminal region of α-synuclein (residues Asp2-Thr92) takes on a α-helical structure and interacts with the detergent micelles, whereas the C-terminal region remains disordered. Additionally, after analysis of the ROA spectrum of a C-terminal truncated variant of α-synuclein, α-synuclein107, a spectrum indicating a higher content of α-helix was found, in line with what one would expect. These observations demonstrate that when a part of α-synuclein becomes ordered, ROA signals originating from these parts are likely to dominate the spectrum.

Finally, to investigate the aggregation propensities of α-synuclein, trifluoroethanol (TFE) was added to the sample mixture, inducing β-sheet-rich fibrils [107,108]. In 5%, TFE solution, α-synuclein adopted ROA spectral features indicative of a mixture of a disordered state and β-sheet (Figure 16). The latter secondary structure is typically characterized by a (narrow) −/+ couplet centered around 1665 cm^−1^, a broad positive band at 1310 cm^−1^ and a broad negative band at 1230 cm^−1^. The β-sheet spectral signatures were more pronounced in a 10% TFE solution, where the beginning of aggregation was observed. It is the central hydrophobic region of α-synuclein termed the non-amyloid-β component (NAC) that participates in the formation of a β-sheet-rich oligomeric state, also responsible for fibril formation and that dominated the ROA spectral intensities.

Very recently, Kurochka and co-workers [109] were able to calculate quantum mechanically the ROA spectra of α-synuclein in its different structural forms. The structures that delivered a good match between the predicted and experimental spectra are in accordance with previous observations. Moreover, their work offered a deeper understanding of specific, yet undescribed, spectral observations. Not only could they extract the structure directly out of the ROA spectra for the first time, but also a clearer picture of the dynamics of α-synuclein could be drawn. Their method for calculating promises the increase in detailed structural analyses of different kinds of pharmaceutically important target proteins by means of ROA.

The two studies combined are an excellent example of how ROA can be used to selectively probe a protein or peptide structure in all kinds of different environments, how the structural properties can be determined, and how the different parts of the compound contribute to the overall ROA spectral intensities.

#### 3.4.2. Diagnostics by Means of ROA

In addition to understanding the structural basis for IDPs and their role in neurodegenerative diseases, another important aspect is the early-stage detection of these. For instance, the predicted rise of patients suffering from Alzheimer’s disease (AD) during the upcoming decades demands a reliable, fast and non-invasive diagnostics method [110]. Although diagnosing AD becomes more and more based on specific biomarkers, clinical (neuropsychological) assessments combined with magnetic resonance imaging or positron emission tomography are often required for making actual reliable diagnoses [111,112,113]. The latter methods are time-consuming, invasive and are unreliable to detect early-stage AD.

Harbatová and co-workers [113] have been investigating the possibility of applying spectroscopic techniques on patient’s blood plasma, a minimally invasive method, to reliably detect AD after having successfully identified the molecular signatures of type 1 diabetes mellitus [114], as well as colon [115] and pancreatic cancer [116]. The principle behind spectroscopic analyses of blood plasma samples for detecting diseases is connected to using biomarkers, while simultaneously circumventing its main limitation of focusing on a single (set of) compound(s). Namely, when recording spectra of blood plasma all biomolecules that might be indicative of AD progression are taken into account in a single spectral signature. By recording ROA, Raman, ECD and IR spectra and using these combined in a statistical evaluation method called linear discrimination analysis, the authors obtained a sensitivity of 94% and a specificity of 83% for the discrimination between the healthy and patient group [113].

**Figure 17:**
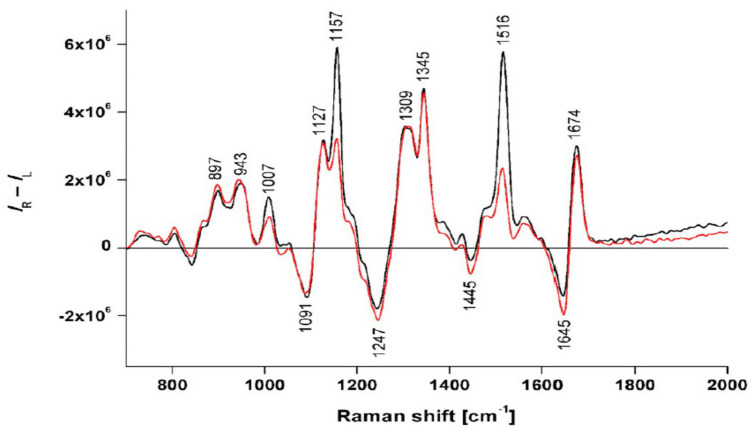
Averaged ROA spectra of blood plasma patients with Alzheimers disease (red) and non-demented elderly controls (black). Reprinted with permission from ref. [113]. Copyright 2019 Elsevier.

As for the ROA spectra in particular, it was found that an α-helical structured protein pattern was observed in blood plasma samples of both groups (vide infra for marker bands) (Figure 17), arising from the dominance of α-helical proteins in blood plasma. Besides minor ROA intensity differences, the largest spectral discrimination between the samples of the two groups was the decrease in carotenoid-associated spectral bands at 1157 and 1516 cm^−1^ for the blood plasma samples of the patient group (Figure 17). The decrease in the concentration of carotenoids is rationalized as the need for eliminating excessive oxidative stress during the development and progression of AD. In similar manners, the ROA technique can be deployed in diagnostics of other diseases and can provide complementary information on the molecular composition of body fluid samples of patients.

## 4. Conclusions

VCD and ROA spectroscopies were developed in the early 1970s, and after a series of technological advancements both techniques have now come of age. VCD in particular has evolved in such a way that it is now accepted by regulatory agencies as proof of AC and has become one of the preferred methods for stereochemical assignments in the pharmaceutical industry. Even though most of these assignments will never be publicized due to confidentiality issues, a significant number of patents list VCD as the main method to determine AC [23]. ROA, on the other hand, has still been mostly limited to academia. Herein, we have presented the underlying principles of each technique, some good practices for their correct use, along with advantages and pitfalls. The examples selected were chosen to demonstrate their strengths in different experimental setups. VCD and ROA offer complementary structural information and, whenever possible, their combination will result in higher confidence assignments. The choice of which technique to use will depend on instrument availability and computational power at hand. VCD is better suited to small-molecules AC assignments in organic solvents, whereas ROA is fully compatible with measurements in aqueous environments of both small-molecules and macromolecules. Additionally, ROA can be used in structural biology and diagnostics as demonstrated in the last example. We hope the present review will stimulate organic and medicinal chemists to consider VCD and ROA as potential tools to unambiguously solve their stereochemical problems.

## Data Availability

Data sharing not applicable.
